# Linking Genetics to Structural Biology: Complex Heterozygosity Screening with Actin Alanine Scan Alleles Identifies Functionally Related Surfaces on Yeast Actin

**DOI:** 10.1534/g3.114.012054

**Published:** 2014-06-17

**Authors:** Stephanie DiPrima, Brian Haarer, Susan Viggiano, Carles Pons, Chad L. Myers, David C. Amberg

**Affiliations:** * Department of Computer Science and Engineering, University of Minnesota, Minneapolis, Minnesota 55455; † Department of Biochemistry and Molecular Biology, SUNY Upstate Medical University, Syracuse, New York 13210

**Keywords:** actin, cytoskeleton, genetic network, myosin, complex heterozygosity

## Abstract

Previous genome-level genetic interaction screens with the single essential actin gene of yeast identified 238 nonessential genes that upon deletion result in deleterious, digenic complex haploinsufficiences with an actin null allele. Deletion alleles of these 238 genes were tested for complex heterozygous interactions with 32 actin alanine scan alleles, which target clusters of residues on the surface of actin. A total of 891 deleterious digenic combinations were identified with 203 of the 238 genes. Two-dimensional hierarchical cluster analysis of the interactions identified nine distinct groups, and the alleles within clusters tended to affect localized regions on the surface of actin. The mutants in one cluster all affect electrostatic interactions between stacked subunits in the long pitch helix of the actin filament. A second cluster that contains the most highly interactive alleles may disrupt the tropomyosin/myosin system, as one of the mutants in that cluster cannot support Type V myosin-dependent movement of secretory vesicles in haploids and causes processivity defects in heterozygous diploids. These examples suggest the clusters represent mutations with shared protein−protein interaction defects. These results show that complex heterozygous interaction screens have benefit for detecting actin-related genes and suggest that having actin filaments of mixed composition, containing both mutant and wild-type subunits, presents unique challenges to the cell.

The genomes of eukaryotic organisms are rich with polymorphisms (Durbin *et al.* 2010) that can attenuate gene activity and underlie inherited phenotypes. To date, geneticists have largely focused on the identification and analysis of Mendelian traits: phenotypes that could be attributed to single genetic loci. However, with the realization that many important human diseases may have complex genetic influences, efforts are beginning to focus on multigenic traits (Zuk *et al.* 2012). Large-scale genetic interaction studies with yeast have enabled the systematic analysis of multigenic influences on phenotype (Tong *et al.* 2001, 2004). Specifically, synthetic genetic array (SGA) analysis enables the systematic construction of all possible haploid double mutant strains. This has led to an unprecedented view of the genetic interaction landscape of a eukaryotic cell (Costanzo *et al.* 2010) and the formulation of rules for the interpretation of the biological meaning of genetic interactions (Dixon *et al.* 2009).

Genetic interactions also can be examined systematically by diploid genetics. In particular, we are interested in the analysis of complex haploinsufficiency (CHI) or the genetic circumstance associated with diploid cells that are heterozygous for null alleles in two (or more) different genes. We suspect that CHI interactions are highly relevant to human genetics, specifically disorders with multigenic influences. For example, simple haploinsufficiency in 32 different transcription factors causes a diverse array of human genetic disorders (Seidman and Seidman 2002), haploinsufficiency of 23 different tumor suppressor genes has been shown to contribute to tumorigenesis (Santarosa and Ashworth 2004), and complex haploinsufficiency has been shown in mouse models to contribute to early aging (Baker *et al.* 2006) and tumorigenesis (Ma *et al.* 2005; Vives *et al.* 2006). Furthermore, recent genome-wide sequencing of 1092 individuals by The 1000 Genomes Project Consortium has shown that on average human individuals inherit ~150 loss-of-function alleles (Abecasis *et al.* 2012). For an individual who inherits 150 loss-of-function alleles, there are 11,175 possibly deleterious digenic CHI interactions, which is not even considering the very large number of single-nucleotide polymorphisms that may partially or completely impact gene function. It is now commonly accepted that the genetic influences on much of human disease is not Mendelian in nature but is genetically complex (Altshuler *et al.* 2008); however, identification of the genetic determinants in genetically complex disorders has been elusive, a problem referred to as “missing heritability” (Queitsch *et al.* 2012) or “phantom heritability” (Zuk *et al.* 2012). We believe that studies in model systems can illuminate aspects of missing/phantom heritability.

CHI interactions have been used to study gene function in *Drosophila* (better known as dominant enhancers) (Raftery *et al.* 1995), worms, and yeast (also known as unlinked non-complementation) (Stearns and Botstein 1988; Welch *et al.* 1993) but this genetic interaction space has been understudied and undersampled. From a genome-wide screen in yeast, we examined a deletion allele of actin (*ACT1)* for CHI interactions with the set of ~4800 deletion alleles of nonessential genes and identified 208 deleterious interactions. The resulting network was highly enriched for actin-related genes as well as others not previously connected to cytoskeletal functions (Haarer *et al.* 2007). Further screening of the nonessential genes has expanded the list of CHI interactions with an actin-null allele to a total of 238 genes (Supporting Information, Table S1). More recently we completed CHI screens with an actin-null allele against deletion alleles for the ~1000 essential genes of yeast and identified an additional 60 CHI interactions. This network was also highly functionally enriched. For example, we found highly significant functional enrichment among the interacting partners for the GO terms “cytoskeleton” (P value = 6.6 × 10^−13^), “TFIID complex” (P value = 1 × 10^−14^), and “proteasome” (P value = 6.6 × 10^−13^) (Haarer *et al.* 2011). In total, that makes nearly 300 deleterious digenic interactions with actin, a result that highlights the potential for CHI interactions to be significant phenotypic drivers in eukaryotic organisms.

Although the yeast actin gene (*ACT1*) displays a rich set of CHI interactions, it may not be representative for all genes. Actin is one of the most functionally diverse proteins in eukaryotic cells; it is directly involved in cell growth, division, polarity, morphogenesis, motility, cytokinesis, endocytosis, exocytosis, and a number of nuclear functions as well (Pruyne and Bretscher 2000a,b; Bettinger *et al.* 2004). This complexity requires that actin physically interact with a great number of proteins, whose genes might be expected to show CHI interaction with actin. In addition, the actin-null allele is haploinsufficient (Deutschbauer *et al.* 2005), and so it represents a highly sensitized background for identifying CHI interactions. Finally, actin exists in two pools, the actin monomer pool and the actin filament pool, with a very rapid and essential exchange between the pools *in vivo*. Therefore, it would be expected that the cytoskeleton should be very sensitive to actin stoichiometry. In fact, experiments suggest actin-haploinsufficient strains are very sensitive to anything that further depletes the monomeric pool through filament stabilization *in vivo* (Haarer *et al.* 2007).

In this study, we addressed the genetic interactions of alleles that cause more subtle effects on actin function. Specifically, we used the actin alanine scan alleles (Wertman and Drubin 1992; Viggiano *et al.* 2010) to screen for CHI interactions with 238 null alleles of nonessential genes confirmed to interact with the actin-null allele. We found that the alanine scan alleles recapitulated subsets of the actin-null allele interactions for a total network of 891 interactions that are correlated structurally to the surface of actin. Two-dimensional clustering of the data identified subsets of alleles with similar interaction profiles that alter spatially congruous regions on actin, which may represent specific protein-protein binding sites.

## Materials and Methods

### Strain construction

The original actin alanine scan allele strains were constructed with both a linked *HIS3* marker and a linked *tub2-201* allele in the β-tubulin gene that confers resistance to benomyl (Wertman and Drubin 1992). We were concerned that the β-tubulin mutation would contribute to the genetic interactions in our screens. Furthermore, in our CHI procedure, we prefer the mutant alleles be marked with a linked nourseothricin resistance (NAT^r^) gene because this gives a very tight selection. Therefore, we undertook reconstructing the actin alanine scan alleles in a more suitable background. During this process, we discovered that seven of the original actin alanine scan mutants had additional mutations. We corrected all seven but found that two mutants previously reported to be recessive lethal alleles were in fact likely dominant lethal alleles and therefore could not be used in our analysis. The correction of these seven alleles and their phenotypic analysis is described in a letter to *Genetics* (Viggiano *et al.* 2010). This left 31 alanine scan alleles, marked with NAT^r^ and without the β-tubulin mutation for CHI analysis. In addition, we included the *act1-159* allele that encodes a filament-stabilizing mutant of actin (Belmont and Drubin 1998). Table 1 lists these alleles, their phenotypes, locations, and number of interactions.

**Table 1:** The actin alanine scan alleles used in this study

Allele	Mutation	Phenotype	Location	CHI Interactions
*act1-101*	D363A,E364A	Ts^−^, recessive	Side	13
*act1-102*	K359A,E361A	Wild type	Side	16
*act1-103*	E334A,R335A,K336A	Lethal, recessive	Front	26
*act1-104*	K315A,E316A	Wild type	Side	18
*act1-105*	E311A,R312A	Cs^−^, Ts^−^, recessive	Front	113
*act1-106*	R290A,K291A,E292A	Lethal, recessive	Side	41
*act1-107*	D286A,D288A	Lethal, partial dominant	Top/bottom	50
*act1-108*	R256A,E259A	Ts^−^, weakly dominant	Back	63
*act1-109*	E253A, R254A	Lethal, partial dominant	Front	52
*act1-110*	E237A,K238A	Lethal, partial dominant	Top/bottom	45
*act1-111*	D222A,E224A,E226A	Ts^−^, recessive	Side	57
*act1-112*	K213A,E214A,K215A	Cs^−^, Ts^−^, recessive	Front	100
*act1-113*	R210A,D211A	Weak Ts^−^, recessive	Front	21
*act1-115*	E195A,R196A	Wild type	Top/bottom	11
*act1-116*	D187A,K191A	Wild type	Back	5
*act1-117*	R183A,D184A	Wild type	Back	4
*act1-119*	R116A,E117A,K118A	Ts^−^, recessive	Back	12
*act1-120*	E99A,E100A	Ts^−^, recessive	Side	7
*act1-121*	E83A,K84A	Cs^−^, Ts^−^, recessive	Side	7
*act1-122*	D80A,D81A	Cs^−^, Ts^−^, recessive	Side	9
*act1-123*	R68A,E72A	Wild type	Back	33
*act1-124*	D56A,E57A	Ts^−^, recessive	Front	11
*act1-125*	K50A,D51A	Cs^−^, Ts^−^, recessive	Side	3
*act1-127*	E270A,D275A	Lethal, recessive	Back	19
*act1-128*	E241A,D244A	Lethal, partial dominant	Top/bottom	40
*act1-129*	R177A,D179A	Ts^−^, recessive	Back	10
*act1-131*	K61A,R62A	Lethal, partial dominant	Top/bottom	54
*act1-132*	R37A,R39A	Cs^−^, Ts^−^, recessive	Back	34
*act1-133*	D24A,D25A	Cs^−^, Ts^−^, recessive	Front	34
*act1-135*	E4A	Wild type	Front	9
*act1-136*	D2A	Wild type	ND	14
*act1-159*	V159N	Ts^−^, recessive	ATP cleft	17

CHI, complex haploinsufficiency; ND, not determined.

Most of the *act1* alanine scan mutant strains were generated as previously described (Haarer *et al.* 2007). Strains carrying the *act1-107*, *-108*, *-127*, *-128*, and *-136* alleles were generated by transforming the corresponding heterozygous diploids previously described (Viggiano *et al.* 2010) with the CEN *URA3ACT1* plasmid pKFW29, followed by tetrad dissection to generate the strains used in the complex heterozygosity screens.

The *act1-159* strain was generated by crossing strain DAY245 (MATa *leu2his3ura3act1-159:Nat^R^tub2-201 lyp1can1*; used for SGA analysis) to BY4741; an *act1-159:Nat^R^* segregant was backcrossed to BY4741 and a haploid *act1-159:Nat^R^* segregant from this diploid was transformed with pKFW29 and used in the complex heterozygosity screens. Unlike the other *act1* strains, this strain carries the *act1*-linked *tub2-201* mutation.

### Complex heterozygosity screens between *act1* alanine scan alleles and the *act1*∆ CHI gene set

The complex heterozygosity screens described in this study were performed as previously described (Haarer *et al.* 2007). To summarize, haploid strains carrying Nat^R^-marked mutant *act1* alleles and also containing *ACT1* on a *CEN URA3* plasmid (pKFW29) were mated to strains deleted (by the kan^R^/G418^R^ marker) for genes previously shown to display complex haploinsufficiency with *act1∆* (Haarer *et al.* 2007) and our unpublished results). Diploids were selected on media containing G418 and nourseothricin, followed by streaking of individual diploid colonies to matched media containing G418 and Nat with or without FOA, which counterselects against cells that carry the *URA3* marker of pKFW29. Streaks on +/− FOA media were incubated at 34.5° and 37° for most crosses, or at 25° and 30° for those strains that display temperature sensitivity due to haploinsufficiency of the particular gene deletion being tested. Scoring of the growth defects is as follows: a score of one is lethality, a score of 2 corresponds to severe growth defects as reflected by small colonies as compared to the control heterozygotes, and a score of 3 reflects colony sizes that are perceptibly smaller than the control heterozygotes.

### Interaction degree feature correlation analysis

The collection of physiological and evolutionary features for the CHI gene set was taken directly from (Costanzo *et al.* 2010). Pearson correlation was used to measure the correlation between the CHI gene degree and each feature using MATLAB.

### Cluster analysis of the complex heterozygous interactions with actin alleles

Actin mutant interaction profiles were clustered using Cluster 3.0 (de Hoon *et al.* 2004). We ran hierarchical clustering for both the alleles and genes sides of the matrix. Similarity was measured using uncentered Pearson correlation with average linkage. The clustering algorithm required the weight of the scores to be reversed: we defined 3 as the strongest score and 1 as the weakest score so that weak scores were closest to non-interactions.

### Molecular modeling

Molecular models were created using UCSF Chimera (Pettersen *et al.* 2004) and Adobe Photoshop.

### Fluorescence microscopy

The Sec4-GFP expressing plasmid pRC556 (gift of Anthony Bretscher) (Schott *et al.* 2002) was transformed into wild-type haploid strain BY4741, *act1-112* haploid strain SVY413, and *act1-112/ACT1^wt^* strain SVY331. Cells were grown to ~2 × 10^7^ cells/mL, spotted on cushions of 25% gelatin in synthetic complete medium on glass slides, and static and time-lapse images were captured on a Zeiss Imager.Z1 epifluorescence microscope with a 100X Plan Apochromator objective (oil, numerical aperture of 1.46) using an Orca ER camera (Hamamatsu Photonics). Images were processed with Zeiss AxioVision software and Adobe Photoshop.

## Results

### Screening for complex heterozygous interactions with the actin alanine scan alleles

We constructed strains carrying 31 different alanine scan alleles (Wertman and Drubin 1992; Viggiano *et al.* 2010) for CHI analysis. In addition, we included the *act1-159* allele that encodes a filament-stabilizing mutant of actin (Belmont and Drubin 1998). We screened this set of actin query alleles against the 238 null alleles of nonessential genes confirmed to show a CHI interaction with an actin-null allele. The resulting digenic complex heterozygotes were then scored for reduced growth rates as previously described using a scoring range of 1−3 corresponding to severe to mild growth defects (Haarer *et al.* 2007). We observed 891 total deleterious interactions with 203 of the 238 genes whose null alleles are CHI with an actin null allele; 35 genes failed to recapitulate a deleterious interaction with any of the actin point mutant alleles despite interaction with the actin null allele (Table S2). Table 1 lists the query alleles, their phenotypes, locations, and number of CHI interactions identified.

To evaluate precision and recall of our CHI data, we repeated CHI analysis for six alleles (*act1-103*, *105*, *112*, *124*, *129*, and *132*) against 9 genes (*RCY1*, *EAF6*, *CNM67*, *GIM3*, *TAF14/ANC1*, *BUD20*, *SHP1*, *SUM1*, and *ARC18*) in quadruplicate. The 26 interactions found across two or more screens were defined as our standard. We then evaluated precision and recall using an independent screen against that standard. Eighteen of the 20 interactions called were found in the standard, yielding a precision of 90%. Eighteen of the 26 interactions in the standard were identified in the independent screen, resulting in a recall of 69%.

**Figure 1:**
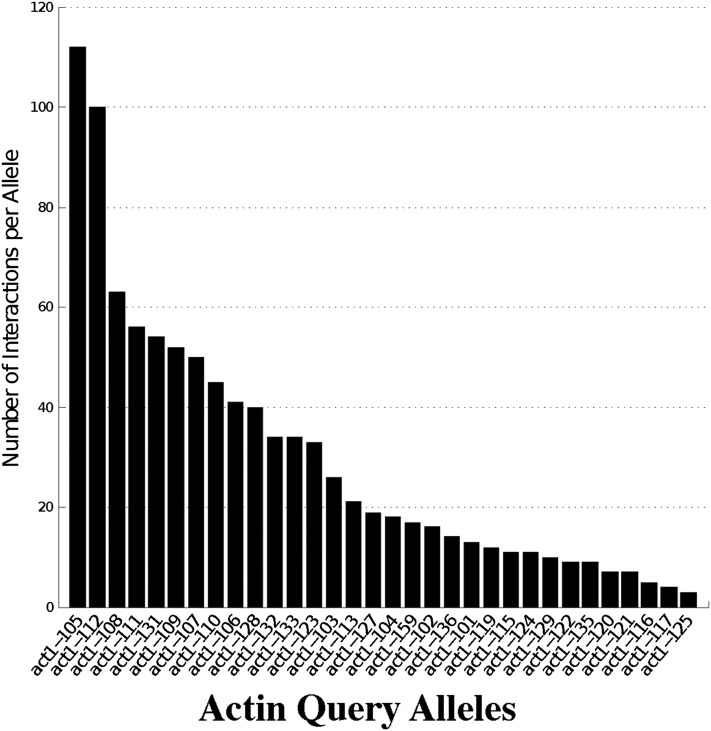
Summary of the number of interactions for each allele. Shown is a bar plot with the number of interactions that each actin alanine scan allele has with the complex haploinsufficiency (CHI) genes. The alleles are sorted on the bottom axis beginning with the greatest number of interactions on the left.

The 31 actin alleles exhibited an average of 29.6 genetic interactions, but most of the alleles displayed a small number of genetic interactions while a few alleles showed a large number of genetic interactions (Figure 1). Like other genetic and physical interaction networks, degree in this network follows a distribution suggestive of a scale-free as opposed to a random network. Similar to haploid genetic interaction degree (Costanzo *et al.* 2010), the mildest alleles (wild-type growth) tended to have lower numbers of interactions (average 13.4) and numbers of interactions tended to increase with the severity of the allele, peaking with the partially dominant lethal alleles (average 48.2). However, it should be noted that in every phenotypic class there are alleles that do not follow these trends. For example, the wild-type allele *act1-123* has 33 interactions and the recessive Cs^-^ Ts^-^ allele *act1-125* had only one interaction.

**Figure 2:**
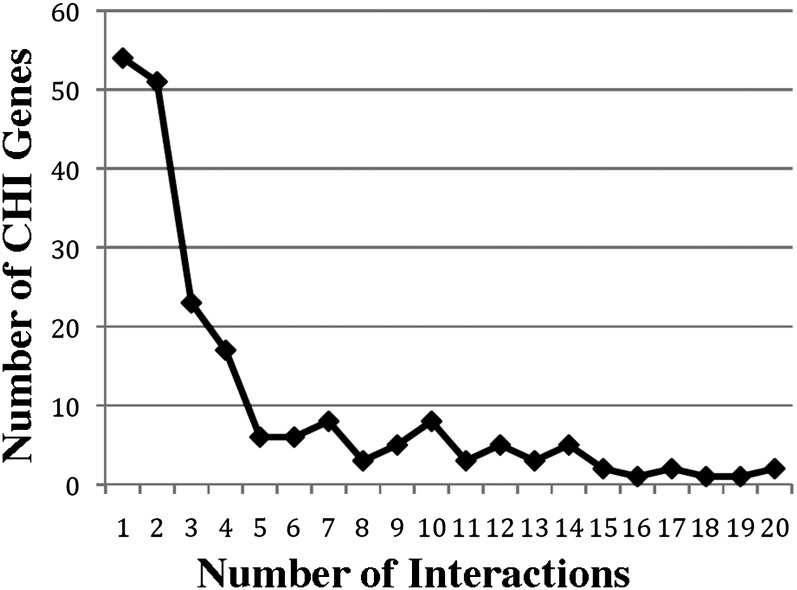
The degree distribution of complex haploinsufficiency (CHI) gene interactions with actin alleles. The number of CHI genes (Y-axis) are plotted *vs.* the number of interactions these genes show with actin alleles (X-axis).

We also examined the distribution of actin allele interactions among the CHI gene set. As mentioned previously, 36 genes failed to manifest a negative epistatic relationship with any of the actin alleles tested. We asked whether the noninteracting genes shared any common features using the web-based FunSpec (Robinson *et al.* 2002). The most notable, statistically significant commonalities were the MIPS Functional Classifications “phosphate metabolism” and “modification by phosphorylation, dephosphorylation, autophosphorylation.” This included the genes: *PTC1*, *PKH1*, *PPH21*, *TPS2*, *RTS3*, *PSR2*, *THI20*, and *RTS1* (P values = 2−3 × 10^−3^). We also examined the distributions of interactions among those CHI genes that interacted with one or more of the alleles tested (see Figure 2). In particular, we compared the number of observed interactions with a number of quantitative physiological and evolutionary properties, as was done for the SGA-derived global haploid genetic interaction network (Costanzo *et al.* 2010) (Table 2). We found that the number of interactions with the actin set was positively correlated with the haploid single mutant fitness defect (Pearson’s r = 0.21, *P* = 0.02). We also found that the number of interactions was positively correlated with chemical-genetic degree (r = 0.22, *P* = 0.009), the number of chemicals which induced single-mutant specific sensitivity in a large screen (Hillenmeyer *et al.* 2008), and phenotypic capacitance (r = 0.27, *P* = 4 × 10^−5^), which reflects variability in actin-based morphological phenotypes in the single mutants (Levy and Siegal 2008). We previously reported that the CHI gene set is enriched for genes whose deletion cause defects in actin organization and cell morphology (Haarer *et al.* 2007).

**Table 2:** Features correlations with CHI degree

Property*^a^*	Correlation Coefficient	P Value	Lower Confidence Interval	Upper Confidence Interval
Yeast conservation	−0.078	0.2417	−0.2061	0.0527
Multi-Functionality	−0.0568	0.3946	−0.1856	0.074
Volatility	−0.0292	0.6622	−0.1588	0.1014
Protein disorder	0.0449	0.5015	−0.0861	0.1744
PPI degree	0.0479	0.4731	−0.0829	0.177
Expression level	0.1234	0.0658	−0.0081	0.2508
Fitness defect	0.2104	0.018	0.0369	0.3717
Chem-gen degree	0.2204	0.0086	0.0572	0.3722
Phenotypic Capacitance	0.2678	4.70E-05	0.142	0.3851

CHI, complex haploinsufficiency; PPI, protein-protein interaction; Chem-gen, chemical-genetic.

a See Costanzo *et al.* (2010) for a complete description of Property categories.

**Figure 3:**
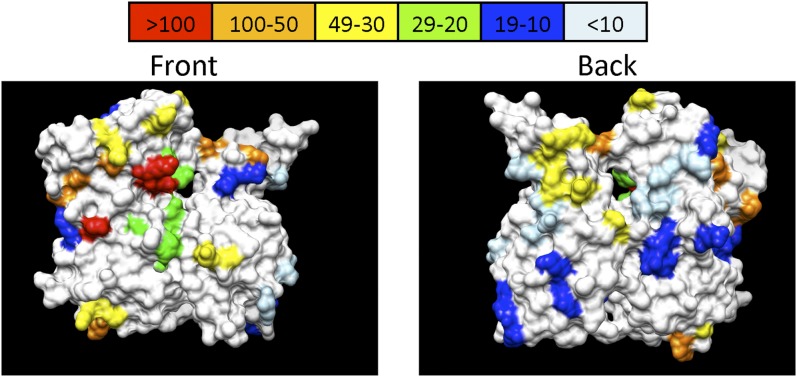
Heat map of Interactions on the surface of actin. This figure shows a heat map of interaction numbers per mutation on the surface of the molecule. The residues altered by alleles with the highest number of interactions are in red and the alleles with the lowest in light blue. Actin was visualized in UCSF Chimera (Pettersen *et al.* 2004).

To determine whether there might be regions on the actin molecule that were particularly sensitive for CHI interactions, we rendered the alanine scan mutants on the surface of actin, color coding the alleles in a heat map fashion by their numbers of interactions (see Figure 3). Because actin forms filaments, we divided the alleles as affecting the following four location groups to measure and compare the interaction levels of each group: the front, the top/bottom, the back, and the sides of the molecule. The front is the area of the molecule that is the most exposed in filaments. The top/bottom area of the molecule affects regions that may be involved in inter-subunit interactions within the long pitch helix and could therefore affect filament stability. The back is largely obscured when actin is in a filament, but mutations here could affect interactions between strands of the filament or could affect monomer-specific functions of actin. The sides line the grooves of the actin filament that are formed between the two strands. In comparison with the front and back, they are smaller in area, are only partially obscured in the filament, and thus could be sites of interaction for both filament and monomer binding proteins. Because some mutations are in the border areas of the molecule, judgment calls were made as to which class the mutation belonged based upon what region of the molecule was most affected by the amino acid changes (all calls listed in Table 1). 

In agreement with our expectations, alleles with exposed mutations in F-actin generally exhibited more interactions than those with changes that were more obscured; however, these differences between the four aforementioned categories were slight and not significant because of a lack of statistical power. We therefore combined the categories into two broader groups: front/top/bottom, which we expected to be highly active because they are exposed or involved in filament stacking and would therefore be more sensitive to changes, and the back/side, which are expected to be tucked inside the filament or otherwise harder to access. The 15 front/top/bottom alleles have a significantly higher median number of interactions than the 17 back/side alleles: front/top/bottom alleles exhibit a median of 37 interactions while the back/side alleles had a median of 11 (*P* < 0.024, Wilcoxon rank-sum test). Note that for this analysis, *act1-159* and *act1-136* were not included because *act1-159* alters residues known to be inside the ATP cleft and therefore is not on the surface of the molecule and *act1-136* alters residues that have not been resolved in crystal structures. In this analysis, the left front of actin appears to be the most active region (see Figure 3, left panel). The binding sites of some important actin binding proteins such as myosin (Lorenz and Holmes 2010), tropomyosin (Behrmann *et al.* 2012), cofilin, and Aip1p (Rodal *et al.* 1999) have been mapped to this region of actin.

### Intrastrand distance measurements correlate with interaction profiles

We hypothesized that the closer the mutations are on the structure, the more their interaction profiles should be correlated. To study this, we examined three sets of distance measurements: monomer, intrastrand “stacked” dimer, and interstrand “backed” dimer. The monomer distance gives the shortest straight-line distance between the alpha carbons of the two nearest mutated amino acids on a single protein. When actin forms filaments, it stacks monomers to form a strand and backs two of those strands together, so we also considered how the mutations may interact in a backed dimer configuration and a stacked dimer configuration. Distances in stacked *vs.* back-to-back dimers also were measured as the shortest straight-line distance between the alpha-carbons of the two nearest mutated amino acids on two different subunits. Pearson correlation was used to measure both the interaction profile similarities and correlations between the distance profiles.

When we compared the interaction profile similarities of the alleles to the distance of the mutated residues on a stacked dimer, we found a significant negative correlation (r = −0.1424, *P* = 0.004). This finding suggests that the smaller the distance between mutations in the stacked dimer, the more similar the genetic interaction profiles tend to be. Backed and monomer distances did not show significant correlations. This may imply that the mutations in general are more detrimental to how actin functions as a filament than as the monomer, and that between stacking and backing, stacking has more influence over the filament stability. In addition, it may suggest that filament-binding proteins are more likely to contact multiple subunits within a filament strand than between filament strands.

### Two-dimensional hierarchical cluster analysis of the complex heterozygous interactions with actin alleles

**Figure 4:**
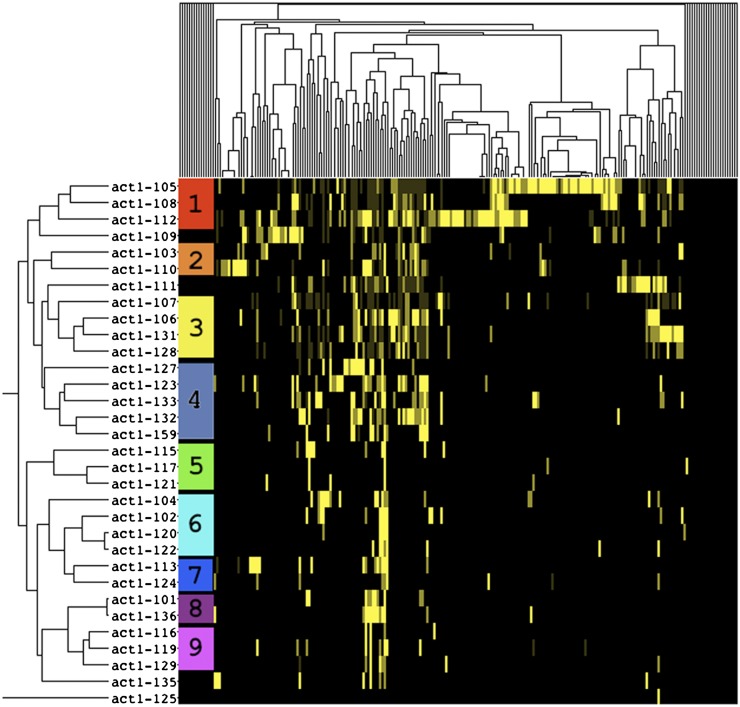
Clustergram of interaction profiles showing allele clustering. This clustergram, visualized using Java Treeview (Saldanha 2004), shows hierarchical clustering of the CHI genes and *act1* alleles. Interactions are shown in yellow (brighter yellow indicates stronger interactions), whereas black is no interaction. Along the left-hand side, clusters of alleles are numbered and designated with a color for reference. The genes are clustered along the top.

We applied hierarchical cluster analysis in two-dimensions (de Hoon *et al.* 2004) to identify patterns of similarity between both the actin alleles and the interacting genes (Figure 4). Similarity was measured using un-centered Pearson correlation with average linkage. For the clustering, strong interactions were given a weight of 3 while the weakest interactions were given a weight of 1, such that the weak scores were closest to non-interactions, shown in black, and the strongest would be the brightest yellow. The actin alleles are indicated and labeled on the left hand side and the CHI gene clusters are unlabeled on the top.

**Figure 5:**
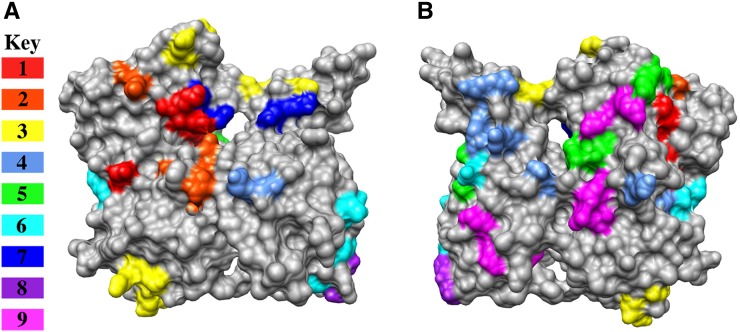
Locations of mutated residues from clustered alleles on the surface of actin. The front (largely exposed in F-actin; A) and the back (largely buried in F-actin; B) surfaces of yeast actin (Vorobiev *et al.* 2003) are rendered in gray and residues mutated by clustered alleles are color coded corresponding to the colors used for these clusters in Figure 4 and as indicated in the key on (A). Rendering was performed with Chimera (Pettersen *et al.* 2004) using the PDB file 1YAG.pdb (Vorobiev *et al.* 2003).

Given the earlier described connection between genetic interaction profile similarity and allele distance, perhaps actin alleles that similarly affect actin structure and function should cluster more closely with respect to their genetic interactions. To test this hypothesis, we defined nine sets of clustered alleles and modeled the locations of the amino acids mutated by these alleles on the structure of actin. The nine clusters are numbered and color-coded on the clustergram shown in Figure 4. Figure 5 shows surface renderings of the actin monomer front (largely exposed in F-actin; Figure 5A) and back (largely buried in F-actin; Figure 5B) with the mutations colored to correspond to the clusters in Figure 4 and shown on the key in Figure 5A. Frequently, the mutated residues of the clustered alleles lie very near each other or affect one domain of the protein. For example, clusters 1 (red) and 2 (orange) affect residues only in subdomain 4 (upper left in Figure 5A), cluster 7 (dark blue) affects residues that are adjacent to each other in subdomain 1, and clusters 5 (green) and 9 (pink) map to bands across the back surface of actin (Figure 5B) which are buried within the filament.

The data suggest that clustered alleles may lead to related structural perturbations. Interestingly *act1-159*, which encodes for a filament stabilizing mutant of actin, is in the large cluster 4 and most of these alleles map to the back of the molecule. The presence of *act1-159* in this cluster suggests that the other alleles in cluster 4 may also cause actin filament stabilization. We have previously noticed that *act1-123* cells have an overly elaborate actin cytoskeleton suggestive of filament stabilization (D. Amberg, unpublished data).

**Figure 6:**
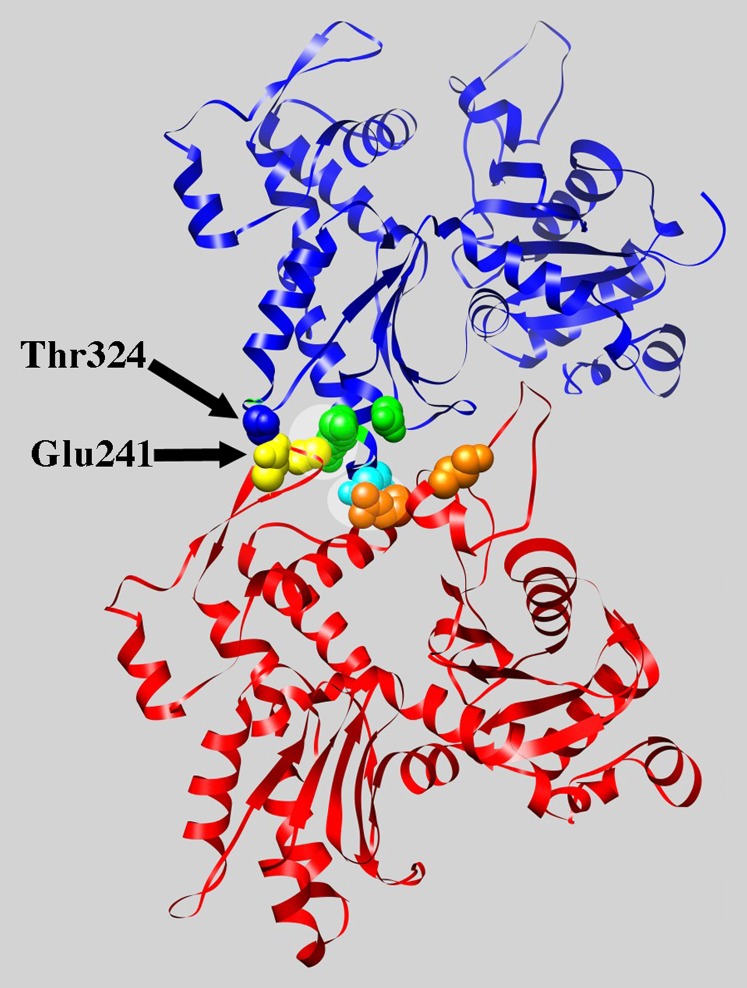
Residues mutated in cluster 3 are predicted to de-stabilize an inter-subunit contact in the long-pitch helix of filamentous actin. Two subunits of the long pitch helix of the filament from a model for rabbit muscle F-actin (Fujii *et al.* 2010) are displayed, one in red and the other in blue. The side chain atoms of residues mutated in the alleles of cluster three have been colored: *act1-106* in green, *act1-107* in cyan, *act1-128* in yellow, and *act1-131* in orange. Note that residues are only colored in the subunit that brings these residues near the intersubunit contact site (*act1-106* and *act1-107* on the blue subunit and *act1-128* and *act1-131* on the red subunit). Thr324 of the blue subunit shown in space-fill (this residue is a serine in yeast actin) and labeled and the two salt-bridges are highlighted. Rendering was performed using the program Chimera (Pettersen *et al.* 2004) and the pdb file 3MFP (Fujii *et al.* 2010).

In the case of cluster 3 (shown in yellow), which appears to affect residues distant on the surface of the actin monomer, the mutated residues map to an important region for inter-subunit contacts within the actin filament (Galkin *et al.* 2008; Oda *et al.* 2009). Figure 6 shows a rendering of two adjacent actin subunits in the long pitch helix from a model of rabbit muscle F-actin (Fujii *et al.* 2010). Each actin subunit is rendered in blue or red and the solvent-exposed surfaces of residues of interest have been rendered and colored. Note in particular that Arg62, mutated in allele *act1-131* (shown in orange on the red subunit), forms a predicted salt bridge with Asp288 mutated in allele *act-107* (shown in cyan on the blue subunit) and Arg290 mutated in allele *act1-106* (shown green on the blue subunit) forms a salt-bridge with Asp244 mutated in allele *act1-128* (shown in yellow on the red subunit). In addition, Glu241 mutated in allele *act1-128* (shown in yellow on the red subunit) is predicted to contact Thr324 (space-filled in blue on the blue subunit; serine in yeast actin). It’s possible that the alleles in cluster 3 group together because they similarly destabilize the long pitch helix of the actin filament which sensitizes the cell to the further loss, through deletion, of a set of functionally related genes.

**Figure 7:**
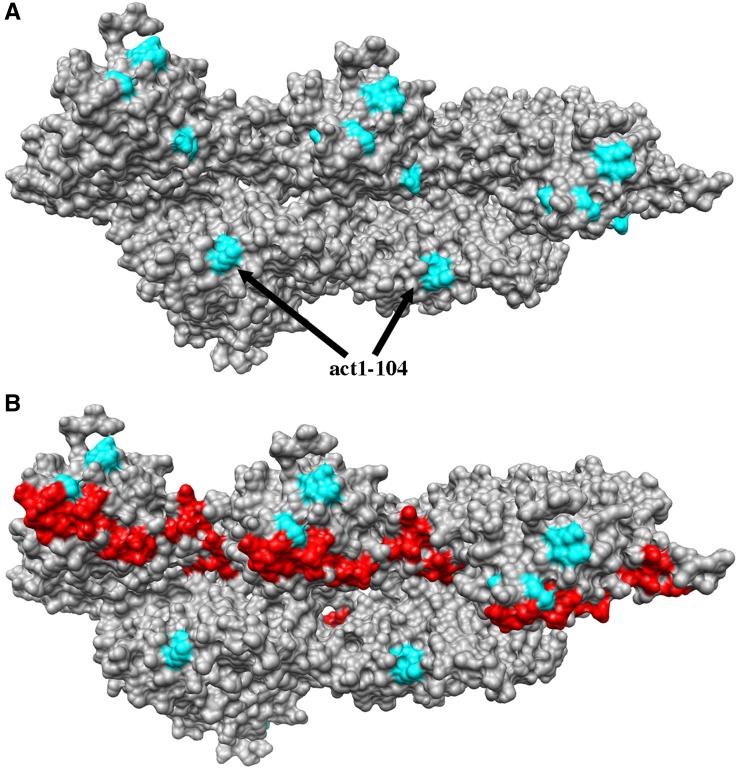
Residues mutated in cluster 6 map to an important site for the binding of actin regulatory proteins tropomyosin and coronin. Five actin subunits from a model for rabbit skeletal muscle actin (Fujii *et al.* 2010) are shown (pdb file 3MFP). Residues mutated by cluster 6 alleles (*act1-104*, *102*, *120*, and *122*) are rendered in cyan (A and B) and predicted contacts with coronin (Galkin *et al.* 2008) are rendered in red (B).

Cluster 6 alters residues on both edges of the actin monomer (cyan in Figure 5, A and B), suggesting that this cluster may also affect actin filament specific interactions. Figure 7 shows the mutated residues of the alleles in cluster 6 (cyan) modeled on 5 subunits of the model for rabbit muscle actin (Fujii *et al.* 2010). These mutations line the groove between the two strands of the actin filament. *act1-104* mutates residues that line the lower edge of the groove (labeled in Figure 7). Cryo-EM models suggest that the important actin regulatory protein coronin binds along this groove and makes extensive contacts with the upper edge shown in red in Figure 7B (Galkin *et al.* 2008), overlapping with the residues mutated by *act1-122* of cluster 6. We cannot say that the shared defects of cluster 6 alleles are caused specifically by defects in coronin binding, but the coronin binding data indicate that this groove is an important site for interactions with actin regulatory proteins.

#### Mixed filament composition and myosin function:

**Figure 8:**
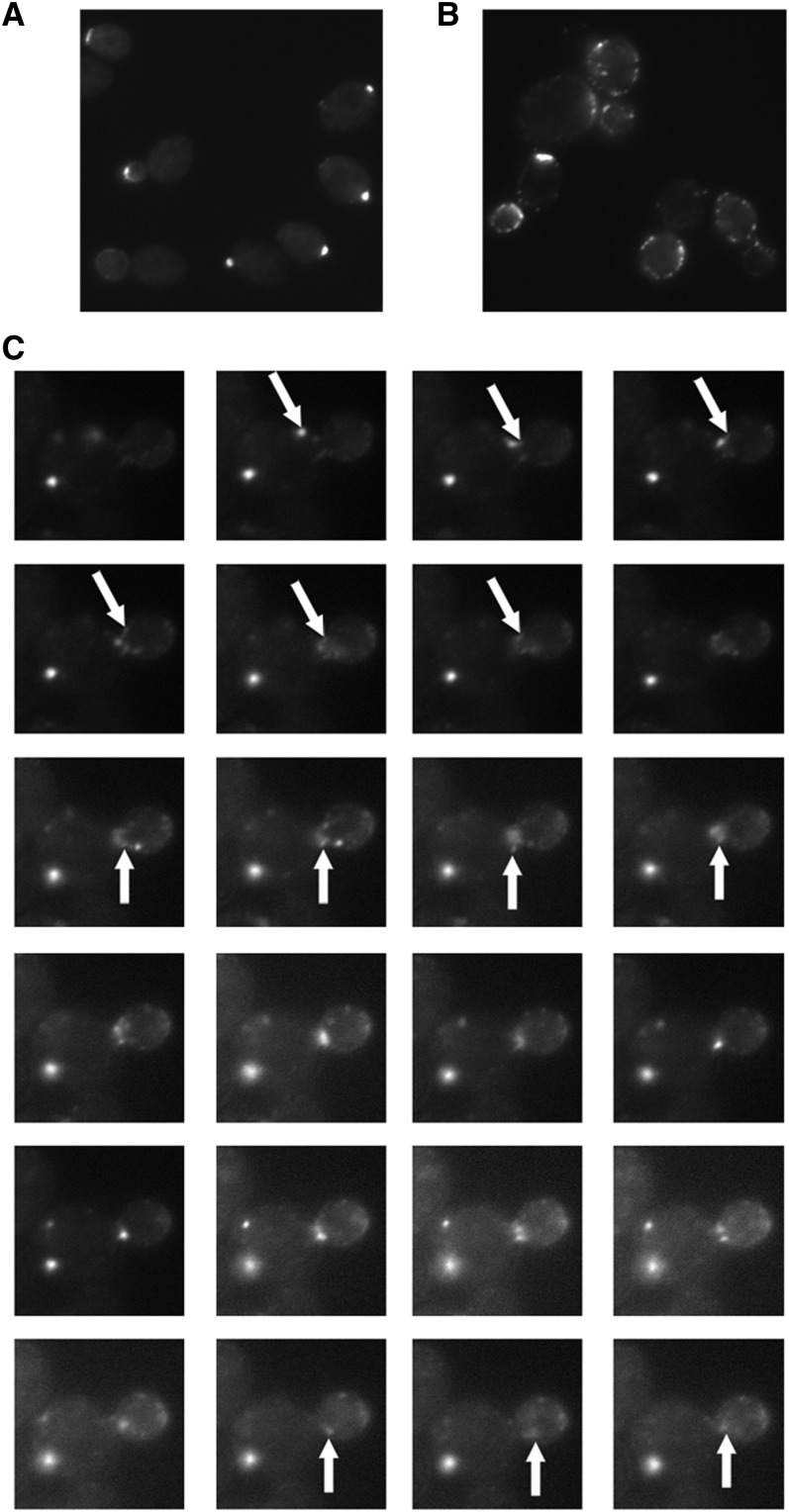
Myo2p-mediated secretory vesicle movement is defective in *act1-112* strains. GFP-Sec4p was expressed in BY4741 wild-type haploid (A), SVY413 *act1-112* haploid (B), and SVY331 *act1-112/ACT1^wt^* heterozygous diploid (C) strains. Static images (A and B) or time-lapse images (C) were captured of green fluorescent protein fluorescence on a fluorescence microscope. Time-lapse images were captured every 1 sec and are in order reading left to right and top to bottom. Events of interest are indicated with arrows.

Preliminary screening for synthetic lethal interactions by SGA with a subset of actin alanine scan alleles used in this study against the 238 genes that have CHI interactions with *act1∆*, showed that most mutants have more synthetic lethal interactions than CHI interactions (C. Boone, personal communication). There is one interesting exception; the *act1-112* allele had 51 CHI interactions but only 4 significant negative SGA interactions with the genes that are CHI with actin. We hypothesize that this allele can in part illuminate the different challenges posed by having uniformly mutant actin filaments *vs.* filaments of a mixed subunit composition. In particular, the myosin system for moving secretory vesicles and organelles into the bud may be sensitive to mixed subunit filaments, which could perturb the binding or processivity of these motors. Focusing specifically on the type V myosin, Myo2p and its directed movement of secretory vesicles into the bud (Pruyne *et al.* 2004), we would expect a haploid mutant defective for Myo2p binding would accumulate nonmotile secretory vesicles. However, in a heterozygous diploid, we would predict that Myo2p vesicles could attach and move toward the barbed end until they encounter a mutant subunit, at which point they would stop and actually appear to move backward due to the rearward subunit flux in the actin cables (Huckaba *et al.* 2006). GFP-Sec4p can be used to characterize Myo2p-mediated secretory vesicle movement (Schott *et al.* 2002). We used this reporter to track vesicle movement in wild-type haploid, haploid *act1-112*, and diploid *act1-112/ACT1^wt^* strains. In wild-type haploid strains we observed accumulation of the GFP-Sec4p at sites of polarization (see Figure 8A), whereas in *act1*-112 haploid (Figure 8B) and *act1-112* heterozygous diploid strains (Figure 8C), we noted nonpolarized spots of vesicle accumulation. In the *act1-112* haploid strains, these spots showed no directed movement (see File S1) but in the *act1-112* heterozygous diploids we could observe bursts of bud-directed movement frequently followed by backward movement (see arrows in Figure 8C and File S2 and File S3; note that File S3 was used to generate the tiled images in Figure 8C). In the *act1-112* haploid the secretory vesicles may be able to eventually find their targets by diffusion and capture whereas in the diploids this mechanism would be confounded by the constant back-tracking of the vesicles; this in turn could lead to more serious defects in the diploids and therefore more genetic interactions.

**Figure 9:**
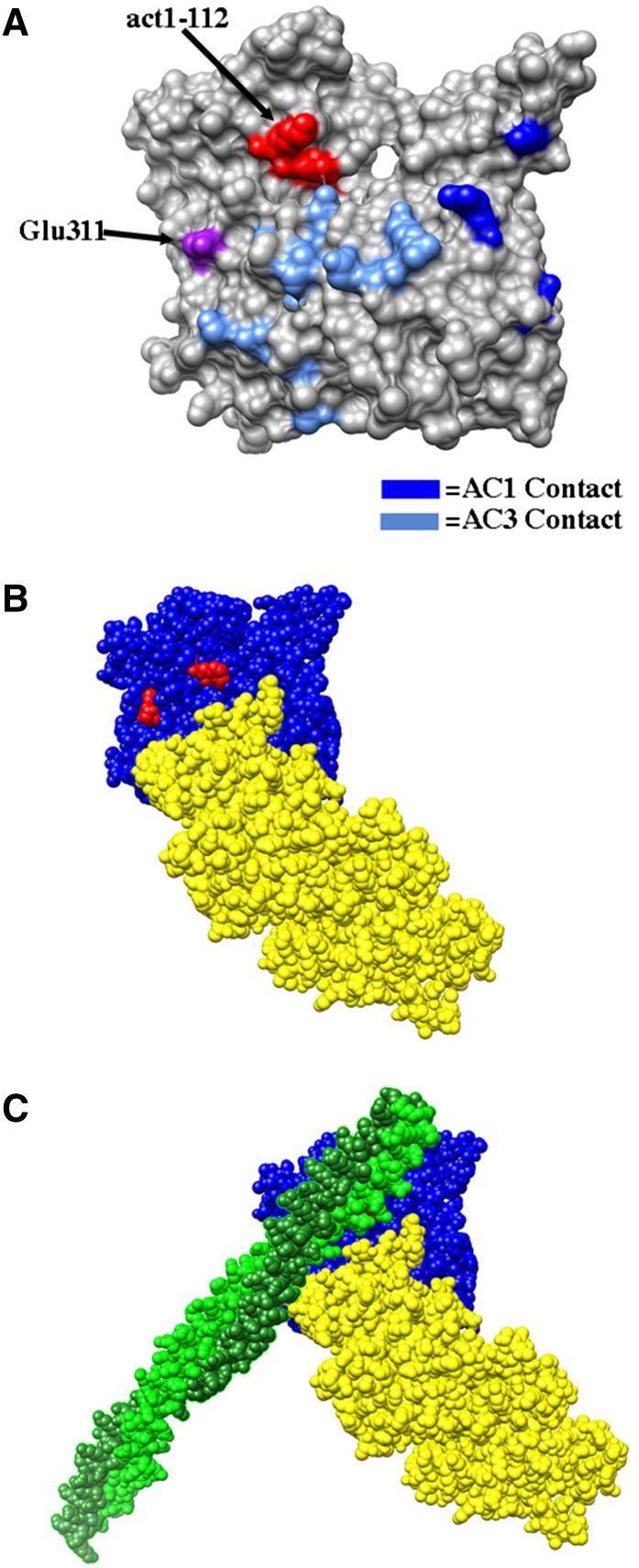
Models of myosin binding to actin. Myosin contact data are rendered based on the Holmes model (Lorenz and Holmes 2010) for the myosin-actin complex on actin pdb file 3MFP.pdb (A) or the Raunser model (Behrmann *et al.* 2012) for the rigor actin-tropomyosin-myosin complex (B and C; pdb file 4A7F). In (A), predicted electrostatic interactions of myosin on actin subunit 1 (AC1) are shown in dark blue, predicted electrostatic interactions of myosin on actin subunit 3 (AC3) are shown in light blue and purple, Glu 311 of actin (mutated in act1-105) is rendered in purple, and the residues mutated in act1-112 are rendered in red. In (B) and (C), actin is rendered in blue, myosin is rendered in yellow, tropomyosin in green (hidden in B), and residues mutated in act1-105 and act1-112 are rendered in red.

It is worth noting that *act1-112* belongs to cluster 1 (red in Figure 4 and Figure 5) with *act1-105* and *act1-108*. In addition, these are the three most highly interactive alleles (see red in Figure 3). If our interpretation of the GFP-Sec4 dynamics in *act1-112* heterozygotes is correct, it suggests that all three mutations may affect myosin binding and/or processivity. Figure 9A shows predicted electrostatic contacts of myosin on actin based on a molecular dynamics/EM-based model for acto-myosin (Lorenz and Holmes 2010). The model predicts that myosin contacts adjacent subunits in the long pitch helix (inter-strand); predicted salt bridges on actin subunit 1 (AC1) are rendered in dark blue and on actin subunit 3 (AC3) in light blue. One of the residues mutated in act1-105 (Glu311 in yeast actin, Asp in muscle actin, rendered in purple) is predicted by the model to make an electrostatic contact with myosin. However, none of the residues mutated in act1-112 (rendered in red) are predicted to contact myosin in this model. More recently, an 8 Å cryo-electron microscopy-derived model for the actin-tropomyosin-myosin rigor complex was published (Behrmann *et al.* 2012). Figure 9, B and C show one actin subunit (blue), one myosin subunit (yellow), and two tropomyosin subunits (green; the tropomyosin subunits are hidden in Figure 9B) from this model. The residues mutated by *act1-105* and *act1-112* are rendered in red. Note that in this model the act1-105 and act1-112 mutated residues actually lie in the tropomyosin-binding site on actin. It has been previously reported that the yeast type V myosins Myo2p and Myo4p are nonprocessive *in vitro* (Reck-Peterson *et al.* 2001), a result that did not agree with the clearly processive behavior of Myo2p *in vivo* (Schott *et al.* 2002). This discrepancy was recently explained by the observation that Myo2p is processive provided the actin filaments are decorated with the tropomyosin isoform Tpm1p (Hodges *et al.* 2012). We hypothesize that the cluster 1 mutants, act1-112 in particular, are defective for either tropomyosin binding and/or myosin binding. In either case, in mixed filaments with wild-type actin, the type V myosins would be able to move on filaments until they encounter subunits defective in myosin binding or lacking tropomyosin due to tropomyosin binding defects.

## Discussion

Sequencing of many individuals within a species has led to a greater realization for the landscape of genetic diversity (Abecasis *et al.* 2012). With this realization comes the challenge to try and predict what combinations of traits contribute to a phenotypic aspect of interest. One approach is to use model systems to systematically construct complex genotypes and look for combinations that influence phenotype. We have reduced the complexity by using yeast to perform whole-genome screens for digenic complex haploinsufficiencies with the single essential actin gene (Haarer *et al.* 2007, 2011), and as reported in this study, digenic complex heterozygosities. The choice of actin as the query arose out of a long-standing interest in the actin cytoskeleton, but we also reasoned that because actin is: 1) important and multifunctional, and 2) moderately haploinsufficient, it should show a rich spectrum of deleterious digenic interactions. In addition, actin function is tightly tied to its ability to exist in a dynamic equilibrium between monomeric and polymeric states and so affecting gene dosage is likely to compromise actin dynamics in unanticipated ways. Indeed the actin CHI space is large: 238 nonessential genes (Haarer *et al.* 2007) and 60 essential genes (Haarer *et al.* 2011). In addition, synthetic dosage lethality screens with actin (1N+1 gene) against the nonessential genes identified 83 interactions (78 negative and 5 positive) and a relatively small overlap with the CHI gene set (Haarer *et al.* 2013). In all of these screens there is extensive functional enrichment, demonstrating that specific functions and processes are sensitive to actin gene dosage.

In this report, we asked a slightly different question: of the 238 genes that are CHI with an actin-null allele, which also display complex heterozygous interactions with actin alanine scan alleles? The actin alanine scan alleles are changes of clusters of charged residues in the primary sequence to alanine (Wertman and Drubin 1992; Viggiano *et al.* 2010). Their purpose was intended to be as genetic reagents to study how proteins interact with the surface of actin and they have been very useful for mapping binding sites on actin (Amberg *et al.* 1995, 1997; Rodal *et al.* 1999; Yuzyuk *et al.* 2002; Clark *et al.* 2006; Clark and Amberg 2007). It is important to point out that there is a fundamental difference between actin complex haploinsufficiency and actin complex heterozygosity; in the first case actin levels are reduced by 1/2 but in the second case actin levels may be normal but filaments of mixed subunit compositions may be formed.

The data set generated in these studies is somewhat unique in a couple of meaningful ways. First of all, it is one of the first networks built from genetic interactions with a large number of alleles of a single gene. It is the first such network whose basis is digenic heterozygous interactions. Finally, it is the first genetic interaction network that can be correlated to defects in protein structure.

Our major hypothesis of this study was that mutations lying near each other on the surface of actin would give similar interaction profiles, reflecting shared phenotypic defects stemming from perturbations of the same protein-protein interactions. We report more than 900 interactions with 32 actin alleles. Using two-dimensional hierarchical cluster analysis we were able to categorize the actin mutants into 9 clusters with similar interaction profiles (Figure 4). Modeling of these clusters on the surface structure of actin was consistent for most clusters as affecting localized regions on actin (Figure 5). However, cluster 3 affects residues on the top and the bottom of the molecule. The mutations in cluster 3 are clearly predicted to disrupt critical stabilizing interactions between stacked subunits in the long pitch helix of the actin filament and therefore cluster 3 identifies an actin-actin binding site that was predicted purely on shared genetic interaction profiles. The mutations in cluster 3 could be affecting actin filaments of mixed composition by four mechanisms: 1) weakening contacts within existing filaments leading to severing, 2) decreasing the on rates at the plus end thereby reducing rates of polymerization, 3) a complete failure to assemble onto the plus end would effectively reduce the amount of polymerizable actin without reducing monomer levels, and 4) by increasing the off rates of subunits from the minus end. In any case, all of these mechanisms would be predicted to lead to the destabilization of actin filaments *in vivo* and apparently in a manner that selectively affects the functions of a shared subset of the CHI genes. Cluster 6 also seems most interpretable in the context of an actin filament. The mutations in cluster 6 line the groove that is formed between the two strands of the long-pitch helix (Figure 7A).

One cluster of particular interest is cluster 1 (red in Figure 5) as this cluster contains the most highly interactive alleles, including one allele (*act1-112*) that appears to be more deleterious in the heterozygous diploid *vs.* haploid context. This suggested to us that these mutants could be affecting the critically important myosin/tropomyosin systems. In yeast there are 5 myosins: 1) Myo1p is a type II myosin that is involved in contraction of the cytokinetic ring; 2) Myo2p is a type V myosin that moves secretory vesicles, vacuoles, golgi, peroxisomes, and the mitotic spindle into the bud; 3) Myo3 and Myo5 are redundant type I myosins that regulate actin assembly in the actin cortical patches; and 4) Myo4p is a second type V myosin that moves mRNA into the bud (Pruyne and Bretscher 2000a,b). In addition yeast has two tropomysosins, Tpm1p and Tpm2p. Tpm1p is the major tropomyosin and it is required for stability of the actin cables which are the tracks used by both type V myosins to move cargo into the bud (Pruyne *et al.* 1998) and is also required for the processivity of Myo2p (Hodges *et al.* 2012). We visualized delivery of Myo2p-dependent cargo in *act1-112* strains by using the secretory-vesicle associated tag GFP-Sec4p (Schott *et al.* 2002). We observed no movement at all in *act1-112* haploids and staccato forward movement followed by backward directed movement in *act1-112* heterozygous diploids. This result is consistent with Myo2p being unable to bind to homogeneous act1-112 filaments and being able to bind but having processivity defects in the heterozygous diploid. Note that there is a rearward flux of actin subunits in actin cables (Huckaba *et al.* 2006). Models are most consistent with the mutations in act1-112 affecting tropomyosin binding (see Figure 9, B and C); however, we do observe actin cables in *act1-112* haploids (data not shown) suggesting that the defects may lie in Myo2p binding; models indicate that the myosin binding site lies very close to the residues affected in act1-112 (see Figure 9A). In sum, the data for clusters 1 and 3 are consistent with the idea that our genetic screen identifies mutations with shared binding defects either because they lie in the binding site or affect the binding site allosterically.

Another hypothesis was that the interacting genes would be clustered by their interactions with actin alleles into groups with shared functions; however, we have not been able to find statistically significant functional enrichment in the gene clusters. One explanation for this could be in the experimental design. The alanine scan alleles were only screened against the genes that are CHI with an actin null allele and so we began with a relatively small set of genes that was already highly enriched for actin-related functions, which could have led to a lack in statistical power to find functional enrichment in the gene clusters. Furthermore, we would have missed genes that would only show allele specific CHI interactions. As a test of this possibility, we screened two alanine scan alleles against the entire nonessential deletion collection and identified several more genes beyond those identified within the subset of 238 genes that are CHI with the null allele. In some cases we found that the new genes were also CHI with the null allele so we can conclude that there are many allele-specific CHI interactors but also that our original CHI screen with the null allele was subsaturating. Both of these issues could have led to a loss in the necessary statistical power to find functional enrichment in the CHI gene clusters. A second possibility that may contribute to the lack in apparent enrichment in the gene cluster might be explained by the multifunctionality of actin. For any particular process or function, actin may influence that process in multiple ways such that the CHI interactions with the actin-null allele reflect multiple inputs into that process. The alanine scan alleles may show specificity for subsets of those inputs as do the interacting genes, resulting in genes with shared functions becoming split by the alleles in the clustergram.

In conclusion, complex heterozygosity screening has clear utility for identifying genes that are relevant to the systems-level functions of the query gene. Our analysis shows that genetic interaction analysis can be used to define functionally related regions on the surface of a protein and that heterozygosity, particularly for polymer systems, can present the cell with unique challenges.

## Supplementary Material

Supporting Information

## References

[bib1] Abecasis G. R. Auton A. Brooks L. D. DePristo M. A. Durbin R. M., 2012 An integrated map of genetic variation from 1,092 human genomes. Nature 491: 56–65 23128226 10.1038/nature11632 PMC3498066

[bib2] Altshuler D. Daly M. J. Lander E. S., 2008 Genetic mapping in human disease. Science 322: 881–888 18988837 10.1126/science.1156409 PMC2694957

[bib3] Amberg D. C. Basart E. Botstein D., 1995 Defining protein interactions with yeast actin *in vivo*. Nat. Struct. Biol. 2: 28–35 7719850 10.1038/nsb0195-28

[bib4] Amberg D. C. Zahner J. E. Mulholland J. M. Pringle J. R. Botstein D., 1997 Aip3p/Bud6p, a yeast actin-interacting protein that is involved in morphogenesis and the selection of bipolar buddin sites. Mol. Biol. Cell 8: 729–753 9247651 10.1091/mbc.8.4.729 PMC276122

[bib5] Baker D. J. Jeganathan K. B. Malureanu L. Perez-Terzic C. Terzic A., 2006 Early aging-associated phenotypes in Bub3/Rae1 haploinsufficient mice. J. Cell Biol. 172: 529–540 16476774 10.1083/jcb.200507081 PMC2063673

[bib6] Behrmann E. Muller M. Penczek P. A. Mannherz H. G. Manstein D. J., 2012 Structure of the rigor actin-tropomyosin-myosin complex. Cell 150: 327–338 22817895 10.1016/j.cell.2012.05.037 PMC4163373

[bib7] Belmont L. D. Drubin D. G., 1998 The yeast V159N actin mutant reveals roles for actin dynamics *in vivo*. J. Cell Biol. 142: 1289–1299 9732289 10.1083/jcb.142.5.1289 PMC2149338

[bib8] Bettinger B. T. Gilbert D. M. Amberg D. C., 2004 Actin up in the nucleus. Nat. Rev. Mol. Cell Biol. 5: 410–415 15122354 10.1038/nrm1370

[bib9] Clark M. G. Amberg D. C., 2007 Biochemical and genetic analyses provide insight into the structural and mechanistic properties of actin filament disassembly by the Aip1p cofilin complex in Saccharomyces cerevisiae. Genetics 176: 1527–1539 17483419 10.1534/genetics.107.072066 PMC1931519

[bib10] Clark M. G. Teply J. Haarer B. K. Viggiano S. C. Sept D., 2006 A genetic dissection of Aip1p’s interactions leads to a model for Aip1p-cofilin cooperative activities. Mol Biol Cell. 17: 1971–1984 16421248 10.1091/mbc.E05-10-0956 PMC1415301

[bib11] Costanzo M. Baryshnikova A. Bellay J. Kim Y. Spear E. D., 2010 The genetic landscape of a cell. Science 327: 425–431 20093466 10.1126/science.1180823 PMC5600254

[bib12] de Hoon M. J. Imoto S. Nolan J. Miyano S., 2004 Open source clustering software. Bioinformatics 20: 1453–1454 14871861 10.1093/bioinformatics/bth078

[bib13] Deutschbauer A. M. Jaramillo D. F. Proctor M. Kumm J. Hillenmeyer M. E., 2005 Mechanisms of haploinsufficiency revealed by genome-wide profiling in yeast. Genetics 169: 1915–1925 15716499 10.1534/genetics.104.036871 PMC1449596

[bib14] Dixon S. J. Costanzo M. Baryshnikova A. Andrews B. Boone C., 2009 Systematic mapping of genetic interaction networks. Annu. Rev. Genet. 43: 601–625 19712041 10.1146/annurev.genet.39.073003.114751

[bib15] Durbin R. M. Abecasis G. R. Altshuler D. L. Auton A. Brooks L. D., 2010 A map of human genome variation from population-scale sequencing. Nature 467: 1061–1073 20981092 10.1038/nature09534 PMC3042601

[bib16] Fujii T. Iwane A. H. Yanagida T. Namba K., 2010 Direct visualization of secondary structures of F-actin by electron cryomicroscopy. Nature 467: 724–728 20844487 10.1038/nature09372

[bib17] Galkin V. E. Orlova A. Brieher W. Kueh H. Y. Mitchison T. J., 2008 Coronin-1A stabilizes F-actin by bridging adjacent actin protomers and stapling opposite strands of the actin filament. J. Mol. Biol. 376: 607–613 18177666 10.1016/j.jmb.2007.12.007 PMC2267021

[bib18] Haarer B. Aggeli D. Viggiano S. Burke D. J. Amberg D. C., 2011 Novel interactions between actin and the proteasome revealed by complex haploinsufficiency. PLoS Genet. 7: e1002288 21966278 10.1371/journal.pgen.1002288 PMC3178594

[bib19] Haarer B. Mi-Mi L. Cho J. Cortese M. Viggiano S., 2013 Actin dosage lethality screening in yeast mediated by selective ploidy ablation reveals links to urmylation/wobble codon recognition and chromosome stability. G3 (Bethesda) 3: 553–561 23450344 10.1534/g3.113.005579 PMC3583461

[bib20] Haarer B. Viggiano S. Hibbs M. A. Troyanskaya O. G. Amberg D. C., 2007 Modeling complex genetic interactions in a simple eukaryotic genome: actin displays a rich spectrum of complex haploinsufficiencies. Genes Dev. 21: 148–159 17167106 10.1101/gad.1477507 PMC1770898

[bib21] Hillenmeyer M. E. Fung E. Wildenhain J. Pierce S. E. Hoon S., 2008 The chemical genomic portrait of yeast: uncovering a phenotype for all genes. Science 320: 362–365 18420932 10.1126/science.1150021 PMC2794835

[bib22] Hodges A. R. Krementsova E. B. Bookwalter C. S. Fagnant P. M. Sladewski T. E., 2012 Tropomyosin is essential for processive movement of a class V myosin from budding yeast. Curr. Biol. 22: 1410–1416 22704989 10.1016/j.cub.2012.05.035 PMC3570268

[bib23] Huckaba T. M. Lipkin T. Pon L. A., 2006 Roles of type II myosin and a tropomyosin isoform in retrograde actin flow in budding yeast. J. Cell Biol. 175: 957–969 17178912 10.1083/jcb.200609155 PMC2064705

[bib24] Levy S. F. Siegal M. L., 2008 Network hubs buffer environmental variation in Saccharomyces cerevisiae. PLoS Biol. 6: e264 18986213 10.1371/journal.pbio.0060264 PMC2577700

[bib25] Lorenz M. Holmes K. C., 2010 The actin-myosin interface. Proc. Natl. Acad. Sci. USA 107: 12529–12534 20616041 10.1073/pnas.1003604107 PMC2906587

[bib26] Ma L. Teruya-Feldstein J. Behrendt N. Chen Z. Noda T., 2005 Genetic analysis of Pten and Tsc2 functional interactions in the mouse reveals asymmetrical haploinsufficiency in tumor suppression. Genes Dev. 19: 1779–1786 16027168 10.1101/gad.1314405 PMC1182340

[bib27] Oda T. Iwasa M. Aihara T. Maeda Y. Narita A., 2009 The nature of the globular- to fibrous-actin transition. Nature 457: 441–445 19158791 10.1038/nature07685

[bib28] Pettersen E. F. Goddard T. D. Huang C. C. Couch G. S. Greenblatt D. M., 2004 UCSF Chimera—a visualization system for exploratory research and analysis. J. Comput. Chem. 25: 1605–1612 15264254 10.1002/jcc.20084

[bib29] Pruyne D. Bretscher A., 2000 a Polarization of cell growth in yeast. J. Cell Sci. 113: 571–585 10652251 10.1242/jcs.113.4.571

[bib30] Pruyne D. Bretscher A., 2000 b Polarization of cell growth in yeast. I. Establishment and maintenance of polarity states. J. Cell Sci. 113: 365–375 10639324 10.1242/jcs.113.3.365

[bib31] Pruyne D. W. Schott D. H. Bretscher A., 1998 Tropomyosin-containing actin cables direct the Myo2p-dependent polarized delivery of secretory vesicles in budding yeast. J. Cell Biol. 143: 1931–1945 9864365 10.1083/jcb.143.7.1931

[bib32] Pruyne D. Legesse-Miller A. Gao L. Dong Y. Bretscher A., 2004 Mechanisms of polarized growth and organelle segregation in yeast. Annu. Rev. Cell Dev. Biol. 20: 559–591 15473852 10.1146/annurev.cellbio.20.010403.103108

[bib33] Queitsch C. Carlson K. D. Girirajan S., 2012 Lessons from model organisms: phenotypic robustness and missing heritability in complex disease. PLoS Genet. 8: e1003041 23166511 10.1371/journal.pgen.1003041 PMC3499356

[bib34] Raftery L. A. Twombly V. Wharton K. Gelbart W. M., 1995 Genetic screens to identify elements of the decapentaplegic signaling pathway in *Drosophila*. Genetics 139: 241–254 7705627 10.1093/genetics/139.1.241 PMC1206322

[bib35] Reck-Peterson S. L. Tyska M. J. Novick P. J. Mooseker M. S., 2001 The yeast class V myosins, Myo2p and Myo4p, are nonprocessive actin-based motors. J. Cell Biol. 153: 1121–1126 11381095 10.1083/jcb.153.5.1121 PMC2174330

[bib36] Robinson M. D. Grigull J. Mohammad N. Hughes T. R., 2002 FunSpec: a web-based cluster interpreter for yeast. BMC Bioinformatics 3: 35 12431279 10.1186/1471-2105-3-35 PMC139976

[bib37] Rodal A. A. Tetreault J. W. Lappalainen P. Drubin D. G. Amberg D. C., 1999 Aip1p interacts with cofilin to disassemble actin filaments. J. Cell Biol. 145: 1251–1264 10366597 10.1083/jcb.145.6.1251 PMC2133144

[bib38] Saldanha A. J., 2004 Java Treeview—extensible visualization of microarray data. Bioinformatics 20: 3246–3248 15180930 10.1093/bioinformatics/bth349

[bib39] Santarosa M. Ashworth A., 2004 Haploinsufficiency for tumour suppressor genes: when you don’t need to go all the way. Biochim. Biophys. Acta 1654: 105–122 15172699 10.1016/j.bbcan.2004.01.001

[bib40] Schott D. H. Collins R. N. Bretscher A., 2002 Secretory vesicle transport velocity in living cells depends on the myosin-V lever arm length. J. Cell Biol. 156: 35–39 11781333 10.1083/jcb.200110086 PMC2173574

[bib41] Seidman J. G. Seidman C., 2002 Transcription factor haploinsufficiency: when half a loaf is not enough. J. Clin. Invest. 109: 451–455 11854316 10.1172/JCI15043 PMC150881

[bib42] Stearns T. Botstein D., 1988 Unlinked noncomplementation: isolation of new conditional lethal mutations in each of the tubulin genes of *Saccharomyces cerevisiae*. Genetics 119: 249–260 3294100 10.1093/genetics/119.2.249 PMC1203409

[bib43] Tong A. H. Evangelista M. Parsons A. B. Xu H. Bader G. D., 2001 Systematic genetic analysis with ordered arrays of yeast deletion mutants. Science 294: 2364–2368 11743205 10.1126/science.1065810

[bib44] Tong A. H. Lesage G. Bader G. D. Ding H. Xu H., 2004 Global mapping of the yeast genetic interaction network. Science 303: 808–813 14764870 10.1126/science.1091317

[bib45] Viggiano S. Haarer B. Amberg D. C., 2010 Correction/completion of the yeast actin, alanine scan alleles. Genetics 185: 391–394 20215471 10.1534/genetics.110.114942 PMC2870974

[bib46] Vives V. Su J. Zhong S. Ratnayaka I. Slee E., 2006 ASPP2 is a haploinsufficient tumor suppressor that cooperates with p53 to suppress tumor growth. Genes Dev. 20: 1262–1267 16702401 10.1101/gad.374006 PMC1472901

[bib47] Vorobiev S. Strokopytov B. Drubin D. G. Frieden C. Ono S., 2003 The structure of nonvertebrate actin: implications for the ATP hydrolytic mechanism. Proc. Natl. Acad. Sci. USA 100: 5760–5765 12732734 10.1073/pnas.0832273100 PMC156274

[bib48] Welch M. D. Vinh D. B. Okamura H. H. Drubin D. G., 1993 Screens for extragenic mutations that fail to complement *act1* alleles identify genes that are important for actin function in *Saccharomyces cerevisiae*. Genetics 135: 265–274 8243992 10.1093/genetics/135.2.265 PMC1205633

[bib49] Wertman K. F. Drubin D. G., 1992 Actin constitution: guaranteeing the right to assemble. Science 258: 759–760 1439782 10.1126/science.1439782

[bib50] Yuzyuk T. Foehr M. Amberg D. C., 2002 The MEK kinase Ssk2p promotes actin cytoskeleton recovery after osmotic stress. Mol. Biol. Cell 13: 2869–2880 12181352 10.1091/mbc.02-01-0004 PMC117948

[bib51] Zuk O. Hechter E. Sunyaev S. R. Lander E. S., 2012 The mystery of missing heritability: genetic interactions create phantom heritability. Proc. Natl. Acad. Sci. USA 109: 1193–1198 22223662 10.1073/pnas.1119675109 PMC3268279

