# Supplementary material for: Linking Genetics to Structural Biology: Complex Heterozygosity Screening with Actin Alanine Scan Alleles Identifies Functionally Related Surfaces on Yeast Actin
Source: G3 (Bethesda). 2014 Jun 17;4(8):1491–501. doi: 10.1534/g3.114.012054 (PMC4132179; doi:10.1534/g3.114.012054)
Supplement: Supporting Information [file supp_4_8_1491__index.html]

Linking Genetics to Structural Biology: Complex Heterozygosity Screening with Actin Alanine Scan Alleles Identifies Functionally Related Surfaces on Yeast Actin — Supporting Information 

# Linking Genetics to Structural Biology: Complex Heterozygosity Screening with Actin Alanine Scan Alleles Identifies Functionally Related Surfaces on Yeast Actin

## Supporting Information for DiPrima *et al.*, 2014

**Files in this Data Supplement:**

- File S1 - GFP-Sec4 vesicles show no directed movement in *act1-112* mutant haploid cells. Time lapse images were captured every 1 sec of GFP-Sec4 fluorescence in *act1-112* haploid strain SVY413 on a Zeiss Imager.Z1 fluorescence microscope. (.mov, 844 KB)
- File S2 - GFP-Sec4 vesicles show non-productive movement in *act1-112/ACT1wt* heterozygous diploid mutant cells. Time lapse images were captured every 1 sec of GFP-Sec4 fluorescence in *act1-112/ACT1wt* diploid strain SVY331 on a Zeiss Imager.Z1 fluorescence microscope. (.mov, 368 KB)
- File S3 - GFP-Sec4 vesicles show non-productive movement in *act1-112/ACT1wt* heterozygous diploid mutant cells. Time lapse images were captured every 1 sec of GFP-Sec4 fluorescence in *act1-112/ACT1wt* diploid strain SVY331 on a Zeiss Imager.Z1 fluorescence microscope. This movie was used to generate Figure 8C. (.mov, 1 MB)
- Table S1 - Genes CHI with *act1Δ*. (.xlsx, 51 KB)
- Table S2 - CHI Interactions (.xls, 88 KB)
